# Permissive versus restrictive temperature thresholds in critically ill children with fever and infection: a multicentre randomized clinical pilot trial

**DOI:** 10.1186/s13054-019-2354-4

**Published:** 2019-03-07

**Authors:** Mark J. Peters, Kerry Woolfall, Imran Khan, Elisabeth Deja, Paul R. Mouncey, Jerome Wulff, Alexina Mason, Rachel S. Agbeko, Elizabeth S. Draper, Blaise Fenn, Doug W. Gould, Abby Koelewyn, Nigel Klein, Christine Mackerness, Sian Martin, Lauran O’Neill, Samiran Ray, Padmanabhan Ramnarayan, Shane Tibby, Kentigern Thorburn, Lyvonne Tume, Jason Watkins, Paul Wellman, David A. Harrison, Kathryn M. Rowan, the FEVER Investigators on behalf of the Paediatric Intensive Care Society Study Group (PICS-SG)

**Affiliations:** 1 Respiratory, Critical Care and Anaesthesia Unit, Paediatric Intensive Care, UCL Great Ormond Street Institute of Child Health, 30 Guildford Street, London, WC1N 1EH UK; 2 Paediatric Intensive Care Unit, Great Ormond Street Hospital for Children NHS Foundation Trust, London, UK; 3 Department of Psychological Sciences, North West Hub for Trials Methodology, University of Liverpool, Liverpool, UK; 4 Clinical Trials Unit, Intensive Care National Audit and Research Centre, London, UK; 5 NHS Foundation Trust, Newcastle, UK; 6 Institute of Cellular Medicine, Newcastle University, Newcastle, UK; 7 Department of Health Sciences, University of Leicester, Leicester, UK; 8 Patient and Parent representative, London, UK; 9 Infection, Inflammation and Rheumatology, UCL Great Ormond Street Institute of Child Health, London, UK; 10 Children’s Acute Transport Service, Great Ormond Street Hospital for Children NHS Foundation Trust, London, UK; 11 Evelina Children’s Hospital, Guy’s and St Thomas’ NHS Foundation Trust, London, UK; 12 Alder Hey Children’s Hospital NHS Foundation Trust, Liverpool, UK; 13 Faculty of Health and Applied Sciences, University of the West of England, Glenside Campus, Bristol, UK

**Keywords:** Sepsis, Infection, Paediatric intensive care, Fever, Paracetamol, Antipyretics, Clinical trial

## Abstract

**Background:**

Fever improves pathogen control at a significant metabolic cost. No randomized clinical trials (RCT) have compared fever treatment thresholds in critically ill children. We performed a pilot RCT to determine whether a definitive trial of a permissive approach to fever in comparison to current restrictive practice is feasible in critically ill children with suspected infection.

**Methods:**

An open, parallel-group pilot RCT with embedded mixed methods perspectives study in four UK paediatric intensive care units (PICUs) and associated retrieval services.

Participants were emergency PICU admissions aged > 28 days to < 16 years receiving respiratory support and supplemental oxygen.

Subjects were randomly assigned to permissive (antipyretic interventions only at ≥ 39.5 °C) or restrictive groups (antipyretic interventions at ≥ 37.5 °C) whilst on respiratory support. Parents were invited to complete a questionnaire or take part in an interview. Focus groups were conducted with staff at each unit. Outcomes were measures of feasibility: recruitment rate, protocol adherence and acceptability, between group separation of temperature and safety.

**Results:**

One hundred thirty-eight children met eligibility criteria of whom 100 (72%) were randomized (11.1 patients per month per site) without prior consent (RWPC). Consent to continue in the trial was obtained in 87 cases (87%). The mean maximum temperature (95% confidence interval) over the first 48 h was 38.4 °C (38.2–38.6) in the restrictive group and 38.8 °C (38.6–39.1) in the permissive group, a mean difference of 0.5 °C (0.2–0.8). Protocol deviations were observed in 6.8% (99/1438) of 6-h time periods and largely related to patient comfort in the recovery phase. Length of stay, duration of organ support and mortality were similar between groups. No pre-specified serious adverse events occurred. Staff (*n* = 48) and parents (*n* = 60) were supportive of the trial, including RWPC. Suggestions were made to only include invasively ventilated children for the duration of intubation.

**Conclusion:**

Uncertainty around the optimal fever threshold for antipyretic intervention is relevant to many emergency PICU admissions. A more permissive approach was associated with a modest increase in mean maximum temperature. A definitive trial should focus on the most seriously ill cases in whom antipyretics are rarely used for their analgesic effects alone.

**Trial registration:**

ISRCTN16022198. Registered on 14 August 2017.

**Electronic supplementary material:**

The online version of this article (10.1186/s13054-019-2354-4) contains supplementary material, which is available to authorized users.

## Background

The fever response has been conserved across animal [1] and even plant species [2] throughout evolution for at least 580 million years [3]. Numerous innate and adaptive immunological processes are accelerated by fever [3, 4]. More rapid recoveries are observed from chickenpox [5], malaria [6] and rhinovirus [7] infections with avoidance of antipyretic medication. The UK National Institute for Health and Care Excellence (NICE) recommend not using antipyretic agents “with the sole aim of reducing body temperature in children with fever.” [8]. There is uncertainty if these immunological advantages of fever outweigh the associated increase in metabolic demand (~ 10% increment in in oxygen consumption per degree centigrade) during critical illness [9].

Observational data in critically ill adults suggest lower mortality with higher peak temperatures in the first 24 h in the context of infection [10]. Randomized clinical trials (RCTs) of antipyretic interventions in critically ill adults are mostly small (26–200 patients) and do not show any consistent effect on early mortality [11–14]. In a recent meta-analysis including eight randomized trials, the relative risk for mortality in sepsis with fever control compared with either no control or a more permissive threshold was 0.93, (95% confidence interval 0.77 to 1.13) [15]. The recent HEAT trial (ACTRN12612000513819) examined the effect of acetaminophen versus placebo to treat fever in 700 critically ill adults with known or suspected infection [9]. Neither mortality nor the number of ICU-free days to day 28 was significantly different. Children differ from adults in both immunity and causes of infection, and hence inferences from adult practice may not be appropriate. We are not aware of any RCTs comparing antipyretics or fever thresholds in critically ill children.

This pilot RCT was conducted to determine the safety and feasibility of a definitive multicentre trial comparing a restrictive approach to fever with a more permissive threshold in critically ill children, with the following objectives: (1) to test the willingness of clinicians to screen, recruit and randomize eligible patients, (2) to estimate the recruitment rate, (3) to test acceptability of the trial, consenting procedures and participant information, (4) to test, following randomization, delivery of, and adherence to the selected temperature thresholds (intervention and control) for antipyretic intervention and to demonstrate separation between the randomized groups in temperature measurements, (5) to inform on the likelihood of major safety concerns, and (6) to inform final selection of a patient-centred primary outcome measure.

The underlying hypothesis is that a more permissive threshold for use of antipyretic interventions is superior to the current restrictive approach.

## Methods

### Trial design and oversight

The FEVER pilot trial was a pragmatic, open, parallel-group multicentre RCT in infants and children accepted for emergency admission to one of four participating paediatric intensive care units (PICUs). The units represented a geographical spread across England and a variety of common configurations for UK PICUs (general or combined general and cardiac units in general academic medical centres or within stand-alone children’s hospitals).

The trial was coordinated and sponsored by the Intensive Care National Audit & Research Centre (ICNARC) Clinical Trials Unit (CTU).

Health Research Authority and research ethics committee (17/LO/1139) approval was obtained. The protocol was registered (ISRCTN16022198) prior to recruitment of the first patient. A trial steering committee, with a majority of independent members, and an independent data monitoring and ethics committee were convened to oversee the trial on behalf of the funder.

The full trial protocol is available in the supplementary appendix.

### Trial population and eligibility criteria

Inclusion criteria were as follows: > 28 days and < 16 years of age, unplanned admission to a participating PICU, fever ≥ 37.5 °C in the first 48 h following contact with the paediatric retrieval service or PICU, new requirement for mechanical ventilation and the treating clinician presumed the cause of the fever was an infective process. Mechanical ventilation was considered to include invasive ventilation, non-invasive ventilation and high-flow humidified oxygen.

Exclusion criteria were as follows: acute encephalopathy, including convulsive status epilepticus; post-cardiopulmonary bypass or known/suspected cardiomyopathy/myocarditis; rhabdomyolysis (a serum creatine kinase concentration at least 10 times the upper limit of normal); malignant hyperthermia, neuroleptic malignant syndrome or drug-induced hyperthermia; receiving palliative care or death perceived as imminent; and previous recruitment to the FEVER pilot trial. These exclusions reflected populations that clinical staff were not in equipoise about the risks and benefits of fever control [16].

### Screening and randomization

Potentially eligible infants and children were screened against the eligibility criteria by the paediatric retrieval team or PICU staff. Randomization took place as soon as eligibility was confirmed, including during transport. Participants were randomly allocated (1:1) via a secure web-based system.

### Trial interventions

Participants received antipyretic interventions at a temperature threshold of either 37.5 °C in the restrictive group or 39.5 °C in the permissive group, until they were no longer receiving any mechanical ventilator support. The 37.5 °C value was selected to represent usual care following extensive feasibility work demonstrating that approximately 60% of emergency PICU admissions receive antipyretic interventions at or below this temperature. The site and technique for temperature measurement and all other care were at the discretion of the treating clinical teams.

### Data collection

A secure, study-specific, web portal was developed containing an electronic case report form. These data were then combined with routinely collected UK Paediatric Intensive Care Audit Network (PICANet; www.picanet.org.uk) data.

### Consent procedures

We employed a “research without prior consent” (RWPC) approach as is appropriate in emergency situations where it is not practically possible to obtain consent prospectively and any delay in commencing treatment allocation may be detrimental [17]. A member of the research team approached the parents/legal representatives as soon as it was possible and appropriate after randomization to discuss the trial, to provide a participant information sheet (supplementary material) and seek consent for continued inclusion in the trial. If the participant was discharged or died prior to their parents/legal representatives being approached, then they were approached by an appropriate team member at a later point either in person or by post with an option to opt out from the trial [17].

### Outcome measures and sample size calculation

The following outcome measures were used to assess the specified objectives. Objective 1: the proportion of eligible patients recruited (target 50%). Objective 2: the number of eligible patients recruited per month (estimated at 6.25 per site per month). Objective 3: staff and parents’ views on the intervention thresholds and trial procedures and the proportion of parents/legal representatives refusing or withdrawing consent. Objective 4: the difference in maximum temperature between the restrictive and permissive groups in the first 48 h, proportion of 6-h time periods in which antipyretic interventions were used as directed in the trial protocol. Objectives 5: occurrence of serious adverse events in each group. Objective 6: characteristics and completeness of potential primary endpoints for a definitive trial including length of PICU stay, length of invasive ventilation, ventilator-free days at day 30, duration of organ support and PICU mortality.

As a pilot RCT, no formal sample size calculations were performed, instead a sample size of 100 was determined to be adequate to estimate candidate patient-centred outcome measures to a necessary degree of precision and to test the trial processes. It was anticipated that this sample size would give 90% power to demonstrate a separation of 0.5 °C in mean peak temperature between temperature groups allowing for 16% withdrawal. 0.5 °C was chosen on the basis of the HEAT trial [9] and our own observational work of the impact of antipyretics on fever in PICU [18].

### Statistical analysis

All analyses were carried out by the randomized group. Continuous variables were summarized as mean (standard deviation) and median (interquartile range) whilst categorical variables were summarized as number (percentage). Analyses were conducted using Stata/SE Version 14.0 (StataCorp LP, College Station, US).

### Parent and staff perspectives

To explore key stakeholder perspectives on trial acceptability, approach to consent and participant information, we invited parents to complete a questionnaire following the FEVER pilot trial recruitment discussion and/or participate in an interview approximately a month after leaving hospital. We also invited staff involved in the pilot trial to take part in a focus group at the end of recruitment. Interviews and focus groups were audio recorded after consent was obtained. Questionnaire and topic guides were developed using previous research [19, 20] and pre-pilot trial qualitative study findings (reported separately) that explored staff and parent perspective on trial design. Qualitative data analysis was thematic and iterative [21]. Interviews continued until no new themes were identified (data saturation) [22]. Quantitative data were analysed using simple descriptive statistics. Data synthesis was pragmatic and drew on the constant comparative approach [23].

## Results

### Objectives 1 and 2: Screening and recruitment rate

**Fig. 1:**
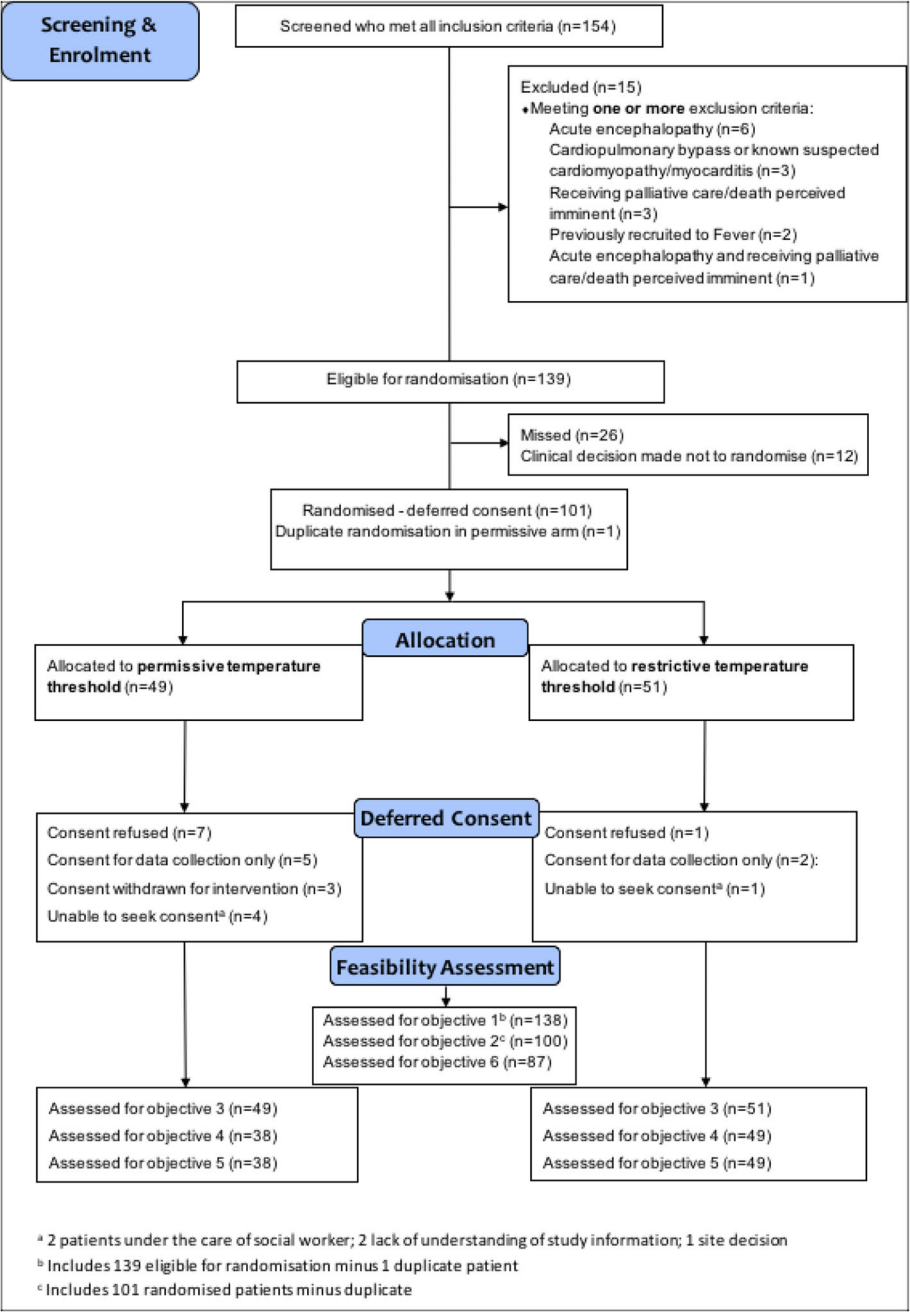
Flow of participants through the pilot randomized clinical trial

Between 25 September and 19 December, 2017, 154 patients were screened and met the inclusion criteria (Fig. 1). Of these, 15 (9.7%) were excluded. Of those eligible, 26 (18.7%) were missed and 12 (8.6%) were not randomized due to local clinical decisions. One hundred one (72.7%) children were randomized into the pilot trial. One patient was removed as a duplicate. 72.5% (100/138) of eligible patients were appropriately randomized. The recruitment rate of 11.1 (95% confidence interval 9.0–13.5) patients per site per month was almost double that estimated from PICANet data.

### Objective 3: Trial acceptability, including consent process and patient information

Of the 100 patients, five (four in the permissive group) were unable to be approached for consent. These included children in the care of social workers and parents who were felt to have insufficient understanding of trial information. Consent to continue in the trial was refused in eight out of the remaining 95 patients. Of these, seven had been allocated to the permissive treatment group. In addition, parents/legal representatives of seven patients (five permissive and two restrictive) refused consent to be treated according to the trial protocol, but consented for ongoing data collection, and a further three patients treated in the permissive group withdrew consent so as to allow an antipyretic intervention to be given. One hospital had seven out of their 10 patients randomized to the permissive group have consent either refused or withdrawn, which accounted for almost half (46.7%) of the total number across all four sites.

Therefore, 87 out of the 100 correctly randomized patients were included in the analysis of objectives 4–6.

A total of 60 parents (49 mothers, 11 fathers) of 57/100 (57%) randomized children took part in a questionnaire (41/60, 68%), an interview (12/60, 20%) or both (7/60, 12%). Eight (six questionnaire and two interview participants) had refused consent for their child’s involvement in the FEVER pilot trial. Forty-eight staff (9/48, 19% medical; 39/48, 81% nursing) participated in one of five focus groups held at the four participating PICUs. All parents interviewed described their support for the trial (see Table 1), which was viewed “an interesting trial” (P30, interview, mother, restrictive), involving a perceived low risk, non-invasive intervention, of which parents were familiar. Many of the questionnaire respondents (32/42, 76%) cited helping other children in the future as their main reason for providing consent, whilst during interviews parents emphasized how they trusted doctors to prioritize their child’s well-being whilst they were in the FEVER pilot trial. Reasons the eight parents provided for declining consent included concerns about their child being in pain or discomfort, increased physiological workload, pre-existing medical condition, risk of epileptic seizures due to family history and incapacity to make an informed decision (see Table 1). Two parents withdrew consent due to concerns about their child being in pain or discomfort during deintensification. Importantly, parents who declined consent to use data already collected, or for their child to continue in the FEVER pilot trial, also described their support for the proposed definitive RCT.

**Table 1:** Staff and parent perspectives from interviews, focus groups and questionnaires by theme

Theme	Subtheme	Illustrative quotations
Trial acceptability	Parent support influenced by the nature of the intervention	“‘I think it’s a brilliant idea, so I am all, I am all for it” (Parent 80, interview mother, permissive). “It’s just how are they gonna give the Paracetamol, when they are gonna give it. I mean if it was more severe, um, more of an invasive study, um, I might have been a bit, I might have had to query it a little bit more but I was happy with, with everything” (Parent 53, interview, father, restrictive).
	Main reasons for consent—trust in doctors/wanting to help others	“If we can find the information to help other children in the future; that would be good” (Parent 35, interview, mother, restrictive). “She’s in a hospital. I mean them people know better than me, so I understand that they would never put a child in harm’s way” (Parent 81, interview, father, restrictive).
	Reasons for declining consent	*Concerns about their child being in pain or discomfort (3 parents)* “We just felt because he could not tell us how he was feeling, like whether he was okay, or whether he was in pain, it was very difficult” (Parent 84, interview, mother, permissive) *Concern about increased workload (1 parent)* “I think allowing her paracetamol to help her temp and heart rate benefits her not tiring” (Parent 68, questionnaire, mother). *Concerns about negative impact due to child’s pre-existing medical conditions (3 parents)* “I think it was, if he had no other underlying medical condition then, and maybe if we had not have been in hospital for 16 weeks previous to that, then possibly, yeah” (Parent 84, interview, mother, permissive). *Risk of seizures due to family history (1 parent)* “My nephew has seizures [..] So we just decided that we just did not want that to even be a possibility” (Parent 84, interview, mother, permissive). *Incapacity and research evidence needed (1 parent)* “At the time he had too much other stuff going on for us to even think about being involved in the study[…] it’s seeing whether there is any research and proof that giving paracetamol straight away is the right thing to do or whether it would go away by itself” (Parent 83, interview, mother, permissive).
	Deintensification as a reason for withdrawing consent	“The only time we eventually pulled him from the trial and gave him paracetamol was when he was awake and he was a lot more distressed, and that was harder for me to watch, especially, especially the way he was, and I said, look, if it’s gonna help, I’d rather you gave him it, but when he was sedated and he was ventilated and everything, he did get a temperature and, like I say, he brought it back down himself” (Parent 49, interview, mother, permissive). “He was withdrawn because, um, he did not actually have a temperature at all and he was in a little bit of pain once he’d been extubated. So they wanted to give him some Paracetamol but he did not have any temperature any way” (Parent 76, interview, mother, permissive).
	Staff concerns and protocol adherence	“I thought it [permissive threshold] was too high because at that stage the patient I was looking after was, was very distressed and very uncomfortable from what I remember I gave paracetamol because I did not fi-, I did not think it was fair on the child to leave them that hot and that distressed” (Staff 01, focus group 3). “we are much happy to, happier to be compliant, erm, if the child was intubated and ventilated and knocked out” (Staff 05, focus group 2).
Research without prior consent	Acceptable in the proposed trial	“I do not think there’s any other way better to go about it” (P79, interview, mother, restrictive). “So many of the trials that we have done over the last few years have worked in the same way, without getting consent and things, it’s actually more normal than, hold on a minute, I cannot do that, we need to get consent first” (Staff 01, focus group 5).

The majority (42/48, 89% of questionnaire respondents) indicated they were satisfied with the FEVER consent process. Four (4/48, 9%) indicated that they were not satisfied with the use of RWPC, although this had not influenced their decision to agree or decline consent. Those who took part in an interview also described how the consent discussion had been well timed and how staff had “clearly explained” (P74, interview, mother, permissive) the trial. Participant information materials were viewed as being “clear and understandable” (P80, interview, mother, permissive).

### Baseline patient characteristics

Overall, the two treatment groups were well matched at baseline (Table 2). Bronchiolitis was the most common diagnosis. Baseline temperature was the same in both groups; however, a greater proportion of patients in the restrictive temperature arm received pre-randomization paracetamol (Additional file 1: Table S3).

**Table 2:** Baseline patient characteristics by treatment group

Variables	Permissive	Restrictive	Total
	*N* = 38	*N* = 49	*N* = 87
Age (years)			
Mean (SD)	1.8 (3.4)	1.1 (2.1)	1.4 (2.7)
Age group (years), *n* (%)			
< 1	24 (63.2)	32 (65.3)	56 (64.4)
1	3 (7.9)	4 (8.2)	7 (8.0)
2–4	5 (13.2)	9 (18.4)	14 (16.1)
5–9	3 (7.9)	3 (6.1)	6 (6.9)
10–15	3 (7.9)	1 (2.0)	4 (4.6)
Gender, *n* (%)			
Female	14 (36.8)	22 (44.9)	36 (41.4)
PIM2r score^a^			
Mean (SD)	0.025 (0.030)	0.025 (0.030)	0.025 (0.029)
Median (IQR)	0.012 (0.008,0.035)	0.012 (0.008,0.037)	0.012 (0.008,0.036)
PIM3 score			
Mean (SD)	0.023 (0.033)	0.025 (0.036)	0.024 (0.034)
Median (IQR)	0.007 (0.005,0.033)	0.007 (0.005,0.038)	0.007 (0.005,0.037)
Source of admission, *n* (%)			
Same hospital	6 (15.8)	13 (26.5)	19 (21.8)
Other hospital	32 (84.2)	36 (73.5)	68 (78.2)
Primary diagnosis, *n* (%)			
Bronchiolitis	19 (50.0)	24 (49.0)	43 (49.4)
Pneumonia/LRTI	9 (23.7)	8 (16.3)	17 (19.5)
Acute respiratory failure	4 (10.5)	7 (14.3)	11 (12.6)
Sepsis/septic shock	2 (5.3)	6 (12.2)	8 (9.2)
Asthma	2 (5.3)	1 (2.0)	3 (3.4)
Seizures/convulsions	1 (2.6)	2 (4.1)	3 (3.4)
Other	1 (2.6)	1 (2.0)	2 (2.3)
Temperature prior to randomization (°C)			
Mean (SD)	38.1 (0.6)	38.1 (0.7)	38.1 (0.6)
Median (IQR)	38.0 (37.7,38.6)	37.9 (37.6,38.3)	37.9 (37.7,38.5)
Type of ventilation, *n* (%)			
High-flow humidified O_2_, *n* (%)	0 (0)	2 (4.1)	2 (2.3)
Non-invasive, *n* (%)	3 (7.9)	3 (6.1)	6 (6.9)
Invasive *n* (%)	35 (92.1)	44 (89.8)	79 (90.8)

*N* total number of patients, *n* number of patients, *SD* standard deviation, *IQR* interquartile range

^a^2016 recalibration

### Objective 4: Protocol adherence and separation between the groups

**Fig. 2:**
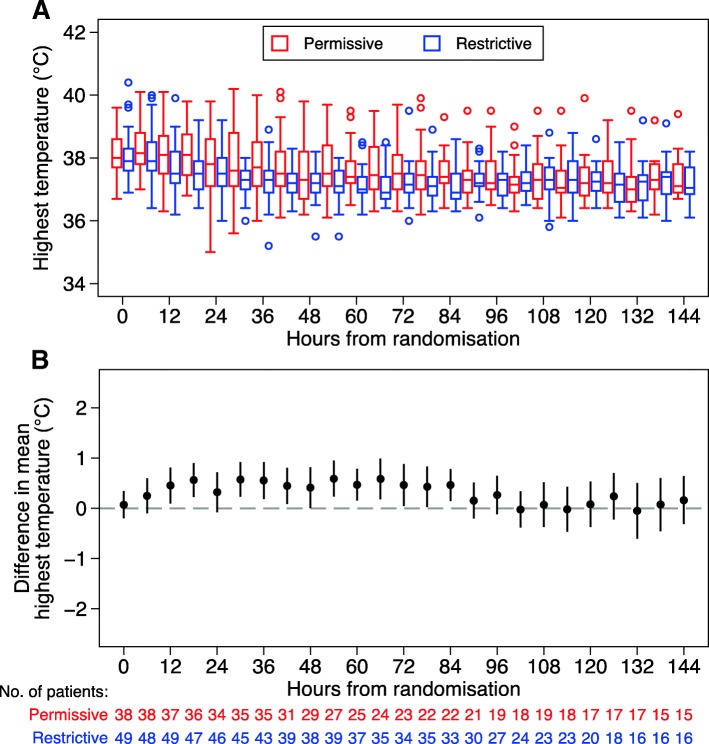
Distribution of peak temperatures in each 6-h period from randomization by treatment group. **a** Median, interquartile ranges and range for each group and **b** between-group differences in means of maximum each 6-h period

Over the first 48 h following randomization, patients in the restrictive group had a mean peak temperature of 38.4 °C (95% confidence interval 38.2–38.6 °C) compared with 38.8 °C (38.6–39.1 °C) in the permissive group. This resulted in a between-group difference in mean peak temperature over the first 48 h of 0.5 °C (0.2–0.8 °C). The separation of around 0.5 °C was maintained up to 84 h following randomization (Fig. 2). More patients received antipyretic interventions in the restrictive group (98%, 48/49) than in the permissive group (50%, 19/38) in the 48 h following randomization (Additional file 1: Table S6).

Protocol deviations were assessed for each patient during each 6-h time period from randomization until death or discharge from PICU. Either not receiving an antipyretic intervention when above the allocated threshold or receiving such an intervention without reaching the threshold were considered deviations. (These definitions are likely to represent overestimates of non-adherence because of interventions in previous, or immediately following, time periods not being considered). Deviations were reported in 39 of 628 6-h time periods (6.2%) in the permissive group and 60 out of 810 time periods (7.4%) in the restrictive group. Overall, 39% of patients in the permissive group and 55% in the restrictive group experienced at least one 6-h period containing a protocol deviation (Table 2).

The embedded qualitative work investigated staff attitudes that may have contributed to non-adherence. Staff had mixed views about the acceptability of the permissive temperature threshold. Focus group participants who did not find the permissive threshold acceptable described how they were unhappy about not administering paracetamol when they thought a child was uncomfortable or in pain. This led to protocol deviations. Suggestions were made to revise the proposed FEVER pilot trial protocol to exclude patients not on mechanical ventilation (e.g. high-flow nasal oxygen) or those close to being extubated when sedation is being weaned.

Overall, the majority of staff described their support for a definitive trial, often stating this trial was needed to provide an answer to an important research question.

“You’re never gonna know the answer if you don’t do a trial” (Staff 04, focus group 2).

### Objective 5: Safety

There were no reported serious adverse events. Three adverse events in total were reported. One seizure was reported in the permissive group (an expected adverse event) that was deemed unlikely to be related to the trial intervention. There were two adverse events in the restrictive group: one seizure that was deemed unrelated and one rhabdomyolysis (an expected adverse event) (Additional file 1: Table S4).

### Objective 6: Potential primary outcome for a definitive trial

For the 86 patients consenting to data collection and having left hospital at the time of data lock, all candidate outcome data were complete on consented patients. Characteristics of the outcome measures are outlined in Table 3, as expected for a pilot trial of this size, there were no significant differences between the groups in any of the outcomes.

**Table 3:** Potential outcome measures by treatment group

Potential outcome measures	Permissive (*N* = 38)	Restrictive (*N* = 49)	Effect estimate (95% CI)	
PICU mortality, *n*/*N* (%)	1/37 (2.7)	1/49 (2.0)	1.3 (0.1, 20.5)^a^	− 0.7 (− 7.2, 5.9)^b^
Hospital mortality, *n*/*N* (%)	1/36 (2.8)	2/46 (4.3)	0.6 (0.1, 6.8)^a^	1.6 (− 6.4, 9.5)^b^
30-day mortality, *n*/*N* (%)	2/37 (5.4)	1/49 (2.0)	2.6 (0.2, 28.1)^a^	− 3.4 (− 11.7, 4.9)^b^
Length of stay in PICU (days)				
All patients	*N* = 37	*N* = 49		
Mean (SD)	7.3 (4.3)	8.1 (8.6)		
Median (IQR)	6 (4, 10)	6 (4, 8)		
Survivors to PICU discharge	*N* = 36	*N* = 48		
Mean (SD)	7.2 (4.3)	7.9 (8.5)		
Median (IQR)	6 (4, 9)	6 (4, 8)		
Receipt and duration of organ support^c^				
Mechanical ventilation, *n* (%)	38 (100)	49 (100)		
Median (IQR)	5 (3, 8)	5 (3, 7)		
Mean (SD)	6.5 (4.6)	6.2 (5.5)	0.3 (− 1.8, 2.5)^d^	
Cardiovascular support, *n* (%)	8 (21.1)	13 (26.5)		
Median (IQR)	2 (2, 3)	3 (2, 6)		
Mean (SD)	0.5 (1.1)	1.2 (2.9)	− 0.7 (− 1.6, 0.2)^d^	
Renal support, *n* (%)	1 (2.6)	3 (6.1)		
Mean (SD)	0.4 (2.4)	0.3 (1.6)	0.1 (− 0.8, 1.0)^d^	
Median (IQR)	*N* < 5	*N* < 5		
Days alive and free (to 28 days) from				
PICU, mean (SD) [*N*]	19.8 (6.4) [37]	20.4 (6.2) [49]	− 0.7 (− 3.4, 2.1)^d^	
Mechanical ventilation, mean (SD)	20.5 (6.7) [38]	21.6 (6.1) [49]	− 1.1 (− 3.8, 1.7)^d^	

*SD* standard deviation, *IQR* interquartile range, *CI* confidence interval

^a^Risk ratio

^b^Absolute risk reduction

^c^Mean and standard deviation reported for all patients, median and interquartile range reported for patients receiving the organ support only (where at least five patients in each group received the support)

^d^Mean difference

## Discussion

In this multicentre, pilot RCT with embedded perspectives study, we investigated the feasibility of conducting a large-scale trial comparing a permissive threshold of antipyretic intervention (≥ 39.5 °C) with a restrictive threshold (≥ 37.5 °C) in critically ill children receiving respiratory support.

We observed that the eligibility criteria were effective in identifying patients and that clinicians were prepared to randomize these patients without seeking prospective informed consent. Our initial estimates of the number of emergency admissions from registry data who met these criteria were shown to be conservative. The rate of recruitment of eligible patients was high with recruitment being completed in approximately half the anticipated time. Parents were overwhelmingly supportive of the RWPC process which is in line with recent findings in the recent Fluids in Shock [20], FIRST-ABC [24] and Oxy-PICU [25] studies.

Nevertheless, there was concern about the acceptability of the permissive protocol to parents and bedside nursing staff. This was manifest in both the number of protocol deviations—albeit against a very strict definition of adherence—and, more importantly, in a high number of instances of consent being declined or withdrawn in the permissive group. Our embedded study revealed that the driver of staff non-adherence and some parents withdrawing consent was concern about patient discomfort or pain, particularly during deintensification with reductions in analgesic and sedative infusions. Staff suggested that only including invasively ventilated children for the duration of intubation would help to minimize these issues.

The variability in consent rates by institution as well as parent and staff views on approaches to consent and trial acceptability will inform our site training for approaching families in a definitive trial.

The protocol achieved a highly significant separation of the groups in terms of observed maximum temperature values especially in the first few days of PICU admission. It is noteworthy that our observed difference in temperature was similar to that achieved in the very much larger HEAT trial of paracetamol or placebo in critically ill adults [9].

Our pilot trial shares weaknesses shared with many trials in critically ill children. Exclusion of cases with acute encephalopathy or congenital cardiac disease limits the generalizability of any findings to a subset of critically ill children. These were felt to be necessary because of our work scoping current practice and equipoise. We did not attempt to control antipyretic therapy prior to PICU referral. We did not attempt to blind clinical staff to the group allocation. Our pragmatic approach meant that clinicians were free to adopt different hemodynamic goals, oxygenation targets or transfusion thresholds that might alter the balance between oxygen delivery and consumption independent of the body temperature thresholds. In addition, as a feasibility trial, we cannot make any conclusions on the effectiveness of a permissive approach to fever.

There are several strengths of FEVER beyond it being the first report of a randomized comparison of permissive and restrictive temperature thresholds in critically ill children. We have demonstrated a high degree of engagement of clinical staff with the protocol across different units and transport teams. The trial processes were largely acceptable to parents and parents. No safety issues were identified and clinical outcomes were readily collected and suitable for a definitive trial.

Although the choice of primary outcome measure for a definitive trial will involve consultation with patients and families and considerations of cost, timings and competing studies, our data permit sample size estimations. For example, a composite outcome of mortality and duration of ventilation with 90% power to detect a 12-h difference in ventilation and no effect on mortality would require a total of around 2000 patients. These pilot data have identified the challenges involved in attempting a definitive trial of fever management in critically ill children. Protocol amendments are likely to improve adherence and retention in a definitive trial but an internal pilot study would be prudent to confirm the impact of these changes.

## Conclusion

The FEVER pilot trial has demonstrated the feasibility of conducting a definitive pragmatic clinical trial of temperature thresholds for antipyretic interventions in critically ill children with some reservations. These are particularly around protocol adherence during recovery. Limiting the intervention to the duration of invasive ventilation may be appropriate.

## Additional file

Additional file 1: **Table S1**. Approach to qualitative data analysis. **Table S2** Protocol deviations by group. **Table S3** Pre-randomization antipyretic interventions by treatment group. **Table S4** Details of adverse events. **Table S5** Number of patients with complete follow-up data for each potential outcome measure. **Table S6** Antipyretic interventions by treatment group. (DOCX 37 kb)
