# Supplementary material for: Permissive versus restrictive temperature thresholds in critically ill children with fever and infection: a multicentre randomized clinical pilot trial
Source: Crit Care. 2019 Mar 7;23:69. doi: 10.1186/s13054-019-2354-4 (PMC6407208; doi:10.1186/s13054-019-2354-4)
Supplement: Supplementary file 1 — Table S1. Approach to qualitative data analysis. Table S2 Protocol deviations by group. Table S3 Pre-randomization antipyretic interventions by treatment group. Table S4 Details of adverse events. Table S5 Number of patients with complete follow-up data for each potential outcome measure. Table S6 Antipyretic interventions by treatment group. (DOCX 37 kb) [file 13054_2019_2354_MOESM1_ESM.docx]

Supplemental Tables

Table S1 Approach to qualitative data analysis

| **Phase** | **Description** |
| --- | --- |
| 1. Familiarising with data | ED and KW read and re-read interview and focus group transcripts noting down initial ideas on themes. |
| 1. Generating initial codes | Initially two complementary data-coding frameworks were developed (one for focus group data (KW), one for interview data (ED) using *a priori* codes identified from the project proposal and topic guides. During the familiarisation stage ED and KW identified additional data-driven codes and concepts not previously captured in the initial coding frame. |
| 1. Developing the coding framework | KW coded 10% of the interview transcripts using the initial coding frame and made notes on any new themes identified and how the framework could be refined. In turn ED coded 10% of the focus group transcripts following the same procedure. |
| 1. Defining and naming themes | Following review and reconciliation by ED and KW, revised coding frames were subsequently developed and ordered into themes (nodes) within the *NVivo* Database. |
| 1. Completion of coding of transcripts | ED completed coding interview transcripts and KW completed coding focus group transcripts in preparation for write-up. |
| 1. Write up | ED and KW developed the manuscript using themes to relate back to the study aims ensuring key findings and recommendations were relevant to the FEVER trial design and site staff training (i.e. catalytic validity) Final discussion and development of selected themes occurred during the write-up phase. [21,26] |

Table S2 Protocol deviations by group

|  | Permissive | Restrictive |
| --- | --- | --- |
|  | (N=38) | (N=49) |
| Receiving antipyretic when temperature below threshold: | | |
| n/N (%) of six-hour periods | 37/589 (6.3) | 20/504 (4.0) |
| n (%) of patients | 14 (36.8) | 11 (22.4) |
| Not receiving antipyretic when temperature above threshold: | | |
| n/N (%) of six-hour periods | 2/39 (5.1) | 40/306 (13.1) |
| n (%) of patients | 2 (5.3) | 20 (40.8) |
| Any protocol deviation: | | |
| n/N (%) of six-hour periods | 39/628 (6.2) | 60/810 (7.4) |
| n (%) of patients | 15 (39.5) | 27 (55.1) |

Table S3

Pre-randomisation antipyretic interventions by treatment group

| Variables | Permissive | Restrictive | Total |
| --- | --- | --- | --- |
|  | N = 38 | N = 49 | N = 87 |
| Any antipyretic intervention, n (%) | 5 (13.2) | 11 (22.4) | 16 (18.4) |
| Paracetamol, n (%) | 3 (7.9) | 9 (18.4) | 12 (13.8) |
| NSAID, n (%) | 0 (0) | 1 (2.0) | 1 (1.1) |
| External and other cooling, n (%) | 3 (7.9) | 3 (6.1) | 6 (6.9) |

N, total number of patients; n, number of patients; NSAID, nonsteroidal anti-inflammatory drug

Table S4 Details of adverse events

| **Treatment group** | **Event** | **Severity** | **Relatedness** |
| --- | --- | --- | --- |
| Permissive | Seizures | Mild | Unlikely |
| Restrictive | Seizures | Mild | None |
| Restrictive | Rhabdomyolysis | Mild | Possibly |

Table S5 Number of patients with complete follow-up data for each potential outcome measure

| Outcome | Permissive | Restrictive |
| --- | --- | --- |
|  | N=38 | N=49 |
| PICU mortality | 37 (97.4) | 49 (100) |
| Hospital mortality | 36 (94.7) | 46 (93.9) |
| 30-day mortality | 37 (97.4) | 49 (100) |
| Length of stay in PICU | 37 (97.4) | 49 (100) |
| Receipt and duration of organ support | 38 (100) | 49 (100) |
| Days alive and free from: |  |  |
| PICU | 37 (97) | 49 (100) |
| Mechanical ventilation | 38 (100) | 49 (100) |

Supplemental Table S6 Antipyretic interventions by treatment group

| Hours^a^ | Permissive Group | | | | Restrictive Group | | | |
| --- | --- | --- | --- | --- | --- | --- | --- | --- |
|  | N^b^ | Paracetamol | NSAID | External and other | N^b^ | Paracetamol | NSAID | External and other |
| Baseline | 38 | 7.9 | 0 | 7.9 | 49 | 18.4 | 2.0 | 6.1 |
| 0 - 6 | 38 | 13.2 | 0 | 13.2 | 49 | 51.0 | 0 | 24.5 |
| 6 - 12 | 37 | 10.8 | 2.7 | 21.6 | 49 | 34.7 | 0 | 22.4 |
| 12 - 18 | 36 | 8.3 | 0 | 5.6 | 47 | 29.8 | 0 | 23.4 |
| 18 - 24 | 35 | 11.4 | 0 | 2.9 | 46 | 34.8 | 0 | 21.7 |
| 24 - 30 | 35 | 8.6 | 2.9 | 5.7 | 45 | 35.6 | 0 | 13.3 |
| 30 - 36 | 35 | 11.4 | 2.9 | 2.9 | 43 | 18.6 | 0 | 11.6 |
| 36 - 42 | 32 | 9.4 | 3.1 | 6.3 | 40 | 20.0 | 2.5 | 2.5 |
| 42 - 48 | 29 | 13.8 | 0 | 3.4 | 39 | 15.4 | 2.6 | 15.4 |
| 48 - 54 | 27 | 14.8 | 0 | 7.4 | 38 | 15.8 | 0 | 21.1 |
| 54 - 60 | 25 | 8.0 | 0 | 0 | 37 | 10.8 | 0 | 2.7 |
| 60 - 66 | 24 | 16.7 | 0 | 0 | 36 | 13.9 | 0 | 8.3 |
| 66 - 72 | 23 | 8.7 | 0 | 13.0 | 34 | 29.4 | 2.9 | 11.8 |
| 72 - 78 | 23 | 4.3 | 0 | 4.3 | 36 | 19.4 | 0 | 11.1 |
| 78 - 84 | 22 | 9.1 | 0 | 0 | 34 | 20.6 | 0 | 8.8 |
| 84 - 90 | 21 | 9.5 | 0 | 4.8 | 32 | 21.9 | 0 | 9.4 |
| 90 - 96 | 19 | 15.8 | 0 | 5.3 | 28 | 25.0 | 0 | 14.3 |
| 96 - 102 | 18 | 11.1 | 0 | 11.1 | 26 | 23.1 | 0 | 19.2 |
| 102 - 108 | 19 | 15.8 | 0 | 5.3 | 24 | 29.2 | 0 | 12.5 |
| 108 - 114 | 18 | 0 | 0 | 0 | 23 | 30.4 | 0 | 8.7 |
| 114 - 120 | 17 | 17.6 | 0 | 0 | 20 | 25.0 | 0 | 15.0 |
| 120 - 126 | 17 | 11.8 | 0 | 0 | 18 | 27.8 | 0 | 11.1 |
| 126 - 132 | 17 | 17.6 | 0 | 0 | 16 | 6.2 | 0 | 12.5 |
| 132 - 138 | 15 | 20.0 | 0 | 0 | 16 | 31.2 | 0 | 18.8 |
| 138 - 144 | 15 | 13.3 | 0 | 0 | 16 | 12.5 | 0 | 12.5 |
